# Efficacy and safety of antiviral treatments for symptomatic COVID-19 outpatients: network meta-analysis and budget impact analysis

**DOI:** 10.3389/fphar.2025.1537018

**Published:** 2025-04-16

**Authors:** Giacomo Berti, Daniele Mengato, Honoria Ocagli, Gianmarco Chinellato, Maria Mazzitelli, Anna Maria Cattelan, Ileana Baldi, Francesca Venturini

**Affiliations:** ^1^ Unit of Biostatistics, Epidemiology and Public Health, Department of Cardiac Thoracic Vascular Sciences and Public Health, University of Padova, Padova, Italy; ^2^ Hospital Pharmacy Unit, Hospital-University of Padua, Padova, Italy; ^3^ Infectious and Tropical Diseases Unit, Hospital-University of Padua, Padova, Italy

**Keywords:** COVID-19, remdesivir, nirmatrelvir-ritonavir, network meta-analysis, outpatient, cost-analysis, antiviral

## Abstract

**Introduction:**

Remdesivir (RDV) and nirmatrelvir/ritonavir (NRM/RTV) are two antiviral agents for treating outpatient adults with mild to moderate symptomatic COVID-19 at high risk of developing a severe disease. The review objectives are to compare the efficacy and safety of these antivirals based on published RCT and real-world data, and to evaluate costs from a healthcare perspective.

**Methods:**

This study provides a network meta-analysis of RDV and NRM/RTV for early treatment of COVID-19. The outcomes analysed were hospitalisation for any cause and serious adverse events. A cost-analysis was performed incorporating drug costs, administration, hospitalisations, and management of adverse events. A budget impact analysis was estimated for the University Hospital of Padua.

**Results:**

Our results indicated that RDV showed a trend towards a lower risk of hospitalisation compared to NRM/RTV (RR 1.59, 95% CI: 0.60–4.20), though this was not statistically significant. For safety, NRM/RTV demonstrated a slightly lower risk of serious adverse events compared to RDV (RR 0.92, 95% CI: 0.31–2.74), but without statistical significance. A cost analysis showed that NRM/RTV could save €550,854.46 per 1,000 patients. Finally, a budget impact analysis based on data from the University Hospital of Padua estimated annual savings of €210,977.25 if all early treatments were administered with NRM/RTV instead of RDV.

**Discussion:**

The comparison of the two antiviral therapies for the early treatment of COVID-19 did not yield statistically significant differences in the potential efficacy and safety to prevent hospitalisation or serious adverse events. However, the results of the cost-analysis showed a saving in favour of NRM/RTV.

## 1 Introduction

Since its discovery in December 2019, severe acute respiratory syndrome coronavirus 2 (SARS-CoV-2) has caused more than 7 million deaths worldwide, according to the World Health Organization (WHO), making it one of the deadliest viruses in human history (COVID-19 Deaths, 2024). Its associated disease, COVID-19, prompted a global response, with significant efforts to develop treatments and preventive measures, particularly vaccines (Dolgin, 2022). These vaccines effectively reduced the severity of illness, hospitalisation rates, and mortality in various populations (Mohammed et al., 2022). Consequently, the COVID-19 emergency officially ended on 5 May 2023 (Assistant Secretary for Public Affairs, 2023).

However, certain populations remain at high risk of developing severe forms of COVID-19. For these vulnerable groups, early antiviral therapies (i.e., administered within 5 days from symptom onset) have been evaluated to prevent progression to severe disease, hospitalisation, and death (Bellino, 2022). In Italy, two antiviral treatments are currently authorised for symptomatic COVID-19 outpatients at high risk of severe disease: intravenous remdesivir (RDV - Veklury^®^) and oral nirmatrelvir/ritonavir (NRM/RTV - Paxlovid^®^) (AIFA, 2023c; 2023b; Bakheit et al., 2023). According to the Italian Medicines Agency (Agenzia Italiana del Farmaco - AIFA), these drugs are indicated for adult patients who do not require additional oxygen and have at least one significant risk factor for severe disease progression. These include active oncological or hematological diseases, chronic kidney or respiratory conditions, immunodeficiencies, obesity, heart and vascular diseases, uncontrolled diabetes, liver disease, haemoglobinopathies, and neurodevelopmental or neurodegenerative disorders (AIFA, 2020). In clinically vulnerable patients, favourable data on the combination of antiviral drugs or antiviral and monoclonal agents emerged (Mazzitelli et al., 2024; Rotundo et al., 2024). Moreover, the efficacy of such antivirals in preventing disease progression was maintained despite the Omicron variants’ lower pathogenicity than the previous ones (Mazzitelli et al., 2023b).

The recommended dosage of RDV in adults and adolescents is a single loading dose of RDV 200 mg administered by intravenous infusion on day 1 and 100 mg administered once daily by intravenous infusion on the following 2 days. Due to the intravenous nature, RDV is usually administered in a hospital or ambulatory setting, prescribed by specialists such as infectious disease clinicians (AIFA, 2023c), which requires a dedicated staff. While RDV was initially approved for inpatient use, it is also indicated for outpatients (adults and pediatric patients weighing at least 40 kg) who do not require supplemental oxygen but are at increased risk of progressing to severe COVID-19 (AIFA, 2023c). On the contrary, NRM/RTV is an oral treatment that can be easily taken at home, with a dose of 300 mg nirmatrelvir and 100 mg ritonavir, twice daily for 5 days (AIFA, 2023b). However, prescribing NRM/RTV requires a prior pharmacological anamnesis to exclude the presence of any interactions with chronic comedications (AIFA, 2023b). Molnupiravir was another drug used to treat COVID-19 disease, whose manufacturer withdrew its application for marketing authorisation in June 2023 (AIFA, 2023a).

Zur et al. (2024) compared RDV, NRM/RTV and Molnupiravir through a network meta-analysis with “hospitalisation” and “adverse events” to measure their difference in efficacy and safety. This network meta-analysis showed that RDV is more effective than NRM/RTV, and there is a trend for greater safety for RDV than NRM/RTV. However, their analysis included studies with heterogeneous outcomes, some of which did not report adverse events, limiting conclusions about treatment safety.

Through a comprehensive network meta-analysis, this article aims to evaluate the evidence on the efficacy and safety of the already available antiviral treatments RDV and NRM/RTV for symptomatic COVID-19 outpatients. Additionally, it provides a budget impact analysis to assess the financial implications of these treatments in healthcare systems, focusing on their cost-effectiveness and overall value in the management of high-risk COVID-19 patients.

## 2 Materials and methods

### 2.1 Data source

This network meta-analysis is based on studies included in the systematic review by Zur et al. (2024), which selected RCTs and observational studies evaluating the efficacy and safety of RDV or NRM/RTV compared to placebo or standard of care. Molnupiravir is excluded from our analysis because its marketing authorization application was withdrawn in June 2023 by the manufacturer, Merck Sharp & Dohme B.V. This means that it is no longer considered an available option for early COVID-19 treatment (Lagevrio, 2023). The outcomes considered were hospitalisation for any reason and serious adverse events for any reason.

### 2.2 Data collection and extraction

Using an electronic form, two reviewers (GB and GC) independently reviewed all studies screened in the systematic review by Zur et al. (2024). Disagreements were resolved by consensus. The following data were extracted: study details (authors, publication year, study design, country), population details (number of participants, study population, age and gender), intervention details (active intervention, duration of treatment), efficacy raw data (number of hospitalisations), and safety raw data (number of serious adverse events).

### 2.3 Risk of bias and publication bias

We used the Cochrane Risk of Bias 2 for randomised trials (RoB 2) (Sterne et al., 2019) to assess the quality of each RCT included in our analysis and the Newcastle-Ottawa Quality Assessment Form (Wells et al., 2000) for cohort studies to assess the quality of each nonrandomised study included in our analysis. Two investigators (GB and GC) reviewed all the studies included and rated them. The Egger test was not applied because the number of studies included was less than 10 (Egger et al., 1997).

### 2.4 Statistical analysis

The primary outcomes of interest were the relative risks (RRs) and 95% confidence interval (CI) of hospitalisation for any cause and serious adverse events for any cause, calculated through network meta-analysis based on a frequentist random-effects model. We used the frequentist network meta-analysis package “netmeta” Version 2.9–0, and R version 4.4. Additionally, we performed a sensitivity analysis to observe differences in efficacy and safety dependent on the study design.

### 2.5 Cost analysis

A cost analysis was conducted to estimate healthcare expenditures associated with two treatment scenarios. In the first scenario (RDV Scenario), 1,000 patients received remdesivir, while in the second scenario (RRM/RTV scenario), 1,000 patients were treated with nirmatrelvir/ritonavir. Given that the choice between RDV and NRM/RTV is primarily determined by patient eligibility (e.g., drug interactions, renal function), we considered the subset of patients who were eligible for both treatments. These were patients who received NRM/RTV (56.28%), as this therapy was generally preferred when both options were available. On the contrary, 43.72% of patients treated with RDV had contraindications to NRM/RTV, making a direct cost comparison inapplicable to them. Therefore, our cost analysis models the economic implications for patients who could have received either treatment. The analysis accounted for various costs, including the ex-factory price, treatment administration expenses, hospitalisation costs, and the management of severe adverse events, all based on real-world healthcare pricing. The ex-factory prices for antiviral treatments were €1,840.00 (AIFA, 2023c) and €1,336.29 (AIFA, 2023b) for RDV and NRM/RTV, respectively. During the COVID-19 epidemic the cost of hospitalisation in Italy was estimated at €8,081.39 (Ferrante, 2024). The median cost of managing a serious adverse event was estimated at €3,725.00 (Tissot et al., 2022).

The intravenous administration process for RDV encompasses three key phases: i) patient preparation, ii) drug preparation, and iii) drug administration. Each phase involves specific activities and sub-activities carried out by healthcare professionals. This structured approach enables direct cost estimation by accounting for the healthcare resources (personnel and drugs) utilised and the time dedicated to each resource as the patient progresses through the care continuum. The model employs a bottom-up (micro-costing) methodology, facilitating a hospital-centric analysis.

Supplementary Table S1 summarises the activities and tasks related to the patient associated with the intravenous administration of RDV. Costs were calculated by multiplying the estimated resource use by the corresponding unit costs. The unit costs for each resource were derived from average hourly gross wages, as outlined in the collective labour agreement of healthcare professionals’ (Supplementary Table S2) (ARAN, 2024). The duration of each phase was estimated by a panel of experts at the University Hospital of Padua, drawing on their practical experience and adhering to therapeutic guidelines (Veklury, 2020).

For “Scenario RDV,” the incidence of hospitalisations and severe adverse events was based on findings from Gottlieb et al. (2022). Consequently, the relative numbers of hospitalisations and severe adverse events for the “Scenario NRM/RTV” were determined using relative risk ratios between RDV and nirmatrelvir/ritonavir (NRM/RTV).

### 2.6 Population for budget impact analysis

The budget impact analysis was based on the number of patients who were eligible for both RDV and NRM/RTV treatment between October 2023 and September 2024. This period is located after the public health emergency for COVID-19 (Assistant Secretary for Public Affairs, 2023), which means that it is not affected by specific health policies aimed at containing SARS-CoV-2 infections. Furthermore, the costs of antiviral treatments for COVID-19 have already been charged to Local Health Agencies, suggesting that the number of early treatments prescribed in subsequent years is unlikely to differ significantly from what was observed during this period. The budget impact analysis conducted for the University Hospital of Padua is limited to this 1-year timeframe, as the pricing for the investigated treatments will be revised on 17 July 2025, for RDV and 15 December 2025, for NRM/RTV (AIFA, 2023b; AIFA, 2023c).

## 3 Results

### 3.1 Study characteristics

We selected eight studies from articles retrieved in the previous systematic review (Zur et al., 2024) (Table 1). Two studies were RCTs: one about RDV (Gottlieb et al., 2022) and one about NRM/RTV (Hammond et al., 2022). Six studies had observational design: one about RDV (Rajme-López et al., 2022) and five about NRM/RTV (Arbel et al., 2022; Wai et al., 2023; Wong et al., 2022; Yip et al., 2023). RDV studies they range of sample size from 126 to 562 patients (Gottlieb et al., 2022; Rajme-López et al., 2022), instead NRM/RTV studies had a range of sample size from 2,246 to 111,588 patients (Hammond et al., 2022; Yip et al., 2023). The patients were enrolled from different countries, more specifically: three studies were from Hong Kong (Wai et al., 2023; Wong et al., 2022; Yip et al., 2023), 1 from Israel (Arbel et al., 2022), 1 from Mexico (Rajme-López et al., 2022), 1 from more countries (United Kingdom, Spain, Denmark and United States) (Gottlieb et al., 2022), 1 from a multicentre database (Ganatra et al., 2023) and 1 from 343 sites around the world (Hammond et al., 2022).

**TABLE 1:** Description of studies included in network meta-analysis.

Author, year	Study design	Country	Treatment	Sample size	N cases	N controls
Arbel et al. (2022)	Observational study	Israel	NRM/RTV	109,254	3,902	105,352
Ganatra et al. (2023)	Observational study	Multicentre	NRM/RTV	111,588	1,131	11,0457
Gottlieb (2022)	RCT	United Kingdom, Spain, Denmark, United States	RDV	562	279	283
Hammond et al. (2022)	RCT	343 Countries	NRM/RTV	2,246	1,120	1,126
Wai et al. (2023)	Observational study	Hong Kong	NRM/RTV	20,339	282	20,057
Rajme-López et al. (2022)	Observational study	Mexico	RDV	126	54	72
Wong et al. (2022)	Observational study	Hong Kong	NRM/RTV	10,525	5,542	4,983
Yip et al. (2023)	Observational study	Hong Kong	NRM/RTV	88,075	4,921	83,154

RCT, randomized clinical trial; RDV, remdesivir; NRM/RTV, nirmatrelvir/ritonavir.

### 3.2 Efficacy and safety of antiviral treatment

**FIGURE 1:**
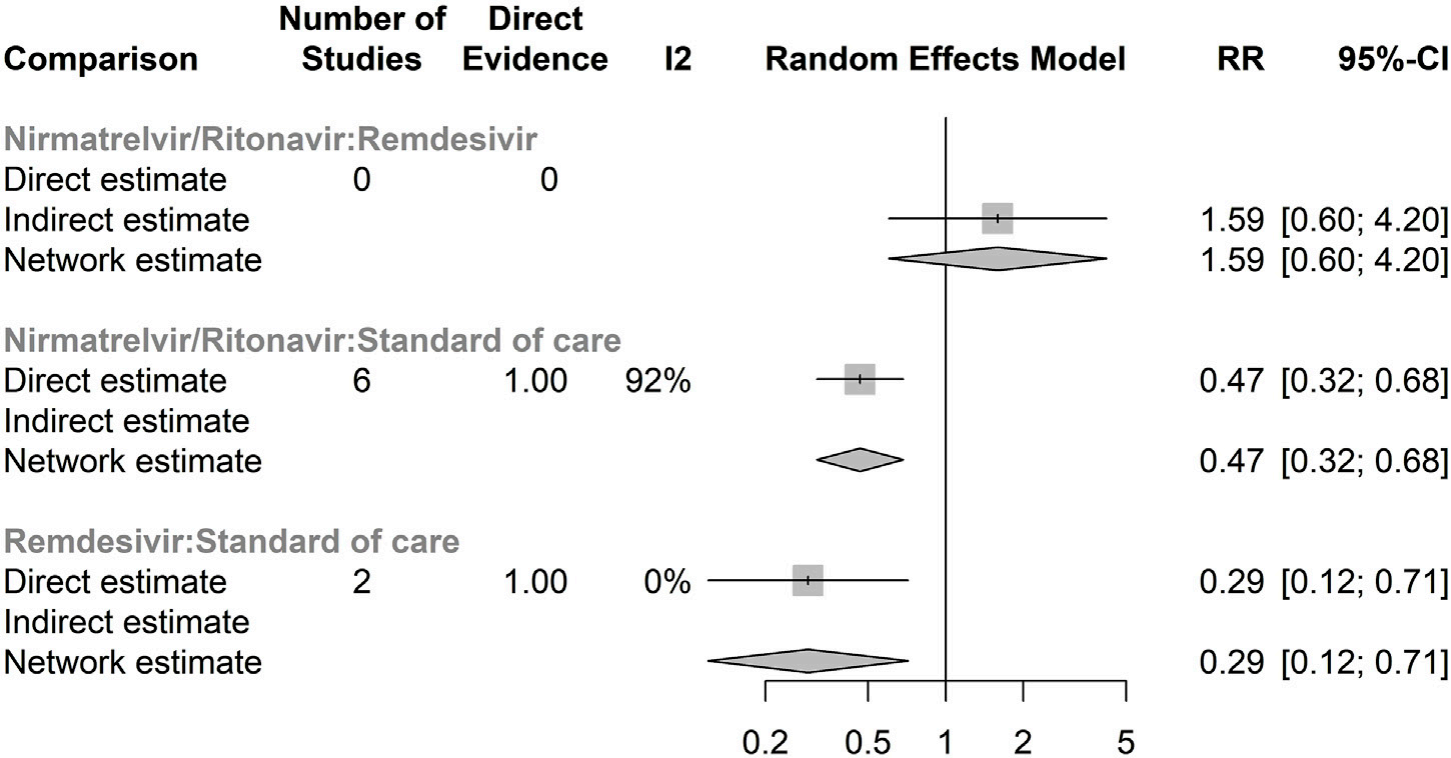
Forest plot of network meta-analysis of hospitalisation for any cause Compared relative risk (RR) for hospitalisation for any cause in different antivirals. The forest plot demonstrates point estimates of risk ratio surrounded by 95% confidence intervals (CI) calculated by random-effects model. Lower and upper confidence limits are presented.

Supplementary Figure S1 illustrates the network meta-analysis graph that evaluates the efficacy of antiviral treatments, explicitly using the RR of hospitalisation for any cause. Notably, there was no direct comparison between RDV and NRM/RTV. All studies included in this updated analysis reported hospitalisation data. Within the antiviral treatment group, 562 hospitalisations were recorded, compared to 4,945 in the control group. The results of our network meta-analysis indicated that remdesivir exhibited a trend toward a reduced risk of hospitalisation compared to NRM/RTV (RR 1.59, 95% CI: 0.60–4.20), though this difference was not statistically significant (Figure 1).

**FIGURE 2:**
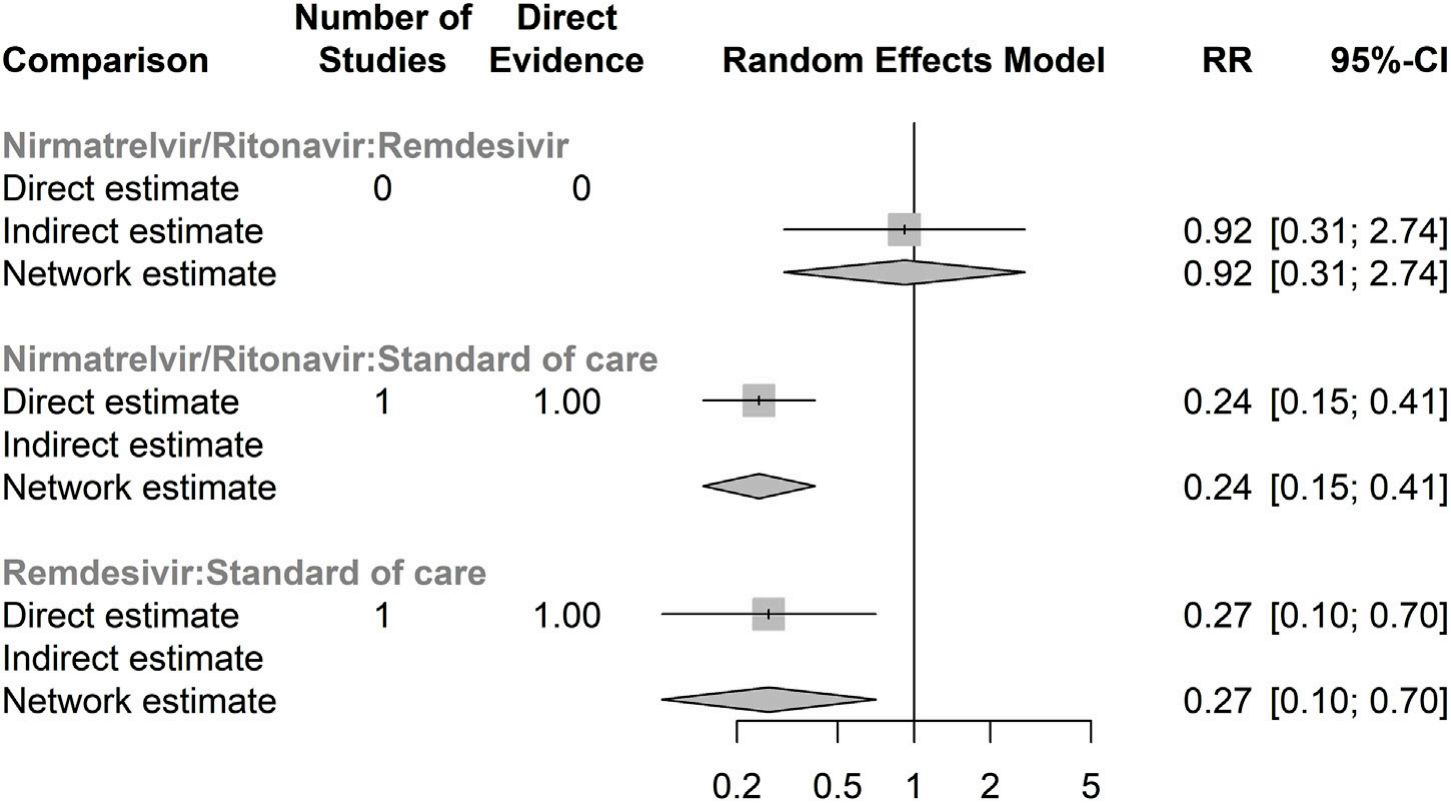
Forest plot of network meta-analysis of serious adverse events for any cause Compared relative risk (RR) for serious adverse events for any cause in different antivirals. The forest plot demonstrates point estimates of risk ratio surrounded by 95% confidence intervals (CI) calculated by random-effects model. Lower and upper confidence limits are presented.

Supplementary Figure S2 presents the network meta-analysis graph for the safety of antiviral treatments, assessed through the RR of serious adverse events for any cause. Similar to the efficacy analysis, there was no direct comparison between RDV and NRM/RTV. A total of 23 serious adverse events were documented in the antiviral treatment group, compared to 93 in the control group. Only two randomised controlled trials (RCTs) provided data on serious adverse events. According to our network meta-analysis, NRM/RTV showed a similar risk of serious adverse events compared to RDV (RR 0.92, 95% CI: 0.31–2.74), although this finding was not statistically significant (Figure 2).

### 3.3 Sensitivity analysis–RCTs versus observational studies

The comparison of RRs for hospitalisation derived from the network meta-analysis, segregated by RCTs and observational studies, revealed divergent trends. In the RCTs subset, NRM/RTV demonstrated a more favourable outcome compared to RDV, with an RR of 0.44 (95% CI: 0.13–1.49) (Supplementary Figure S3). Conversely, when focusing on observational studies, the trend reversed, indicating a decreased risk for hospitalisation for RDV (RR 1.85, 95% CI: 0.56–6.15) (Supplementary Figure S4). However, neither of these measures reached statistical significance.

### 3.4 Cost analysis

The duration of RDV administration is 65 min and it costs €25.73. The cycle of RDV treatment is composed of three consecutive days. Hence, the total cost of RDV administration is €77.19.

Taking into account the incidence of hospitalisation for any cause and serious adverse events for any cause measured by Gottlieb et al. (2022), in “Scenario RDV” the estimated costs of treatment, hospitalisation and serious adverse events are €1,916,140.00, €58,186.01 and €66,677.50 respectively. Instead, in “Scenario NRM/RTV” the estimated costs of treatment, hospitalisation and serious adverse events are €1,336,290.00, €92,515.75 and €61,343.30 respectively (Table 2). Hence, the estimated total costs were €2,041,003.51 for “RDV Scenario” and €1,490,149.05 for “NRM/RTV Scenario”, with a range from €1,391,871.63 to €1,763,367.58 if it was considered the best and worst RRs of NRM/RTV in comparison to RDV for hospitalisation and serious adverse events outcomes. Finally, “NRM/RTV Scenario” could save €550,854.46 (range from €649,131.88 to €277,635.92 considering the best and worst RRs) than “RDV Scenario” each 1,000 patients in early treatment for COVID-19.

**TABLE 2:** Costs of 1,000 patients treated with remdesivir or nirmatrelvir/ritonavir. Stratified by treatment, hospitalisation and serious adverse events.

Costs	Remdesivir	Nirmatrelvir/Ritonavir	Difference
Treatment	1,916,140.00 €	1,336,290.00 €	579,850.00 €
hospitalisation for any cause	58,186.01 €	92,515.75 €	−34,329.74 €
Serious adverse event	66,677.50 €	61,343.30 €	5,334.20 €
Total	2,041,003.51 €	1,490,149.05 €	550,854.46 €

The costs of hospitalisation and serious adverse events were estimated through the incidence of events in Gottlieb et al. for remdesivir, and the RRs estimated from network meta-analysis for nirmatrelvir/ritonavir.

### 3.5 Budget impact analysis

At the University Hospital of Padua, from October 2023 to September 2024, there were 493 patients eligible for both treatments. Applying the costs analysed in the “NRM/RTV Scenario”, administering NRM/RTV instead of RDV to all patients receiving early treatment for COVID-19 would save €271,571.25 annually compared to the cost of treating all patients with RDV.

### 3.6 Assessment of risk of bias

The RCT of NRM/RTV (Hammond et al., 2022) was considered to have a low risk of bias, instead the RCT of RDV (Gottlieb et al., 2022) had some concerns in the randomisation process. The RoB2 results of for RCTs are summarised in Supplementary Figure S5. All included cohort studies were classified as good quality based on the Newcastle-Ottawa Quality Assessment method; however, three studies (Arbel et al., 2022; Wai et al., 2023; Yip et al., 2023) had some concerns regarding the comparability parameter, one study (Ganatra et al., 2023) had some concerns about the selection parameter and one study (Yip et al., 2023) had some concerns about the outcome parameter. The results of the risk of bias for observational studies are summarised in Supplementary Table S6.

## 4 Discussion

The cost-effectiveness analysis between these two treatments in this specific setting was conducted to assess whether the superior efficacy of RDV over NRM/RTV, as reported by Zur et al. (2024), could translate into cost savings despite its higher cost and intravenous administration. Unlike Zur et al., our network meta-analysis includes only studies comparing RDV and NRM/RTV to the standard of care, excluding those on Molnupiravir. Additionally, our network meta-analysis focuses solely on serious adverse events for therapy’s safety, while Zur et al. (2024) considered all adverse events. This methodological distinction may explain why we did not observe a statistically significant difference in hospitalisation risk between RDV and NRM/RTV, whereas Zur et al. found RDV to be superior. At the University Hospital of Padua, our findings suggest that both antivirals are more effective than standard of care, analysing hospitalisation events in predominantly vaccinated populations (Mazzitelli et al., 2023b; 2023a).

In real-world clinical practice, the choice between RDV and NRM/RTV for early COVID-19 treatment is primarily dictated by patient-specific factors, particularly concomitant chronic medications and comorbidities. Among the two, NRM/RTV has stricter eligibility criteria due to its well-documented drug-drug interactions, which frequently made RDV the only feasible option. However, in cases where both treatments were viable, NRM/RTV was overwhelmingly preferred by physicians due to its oral administration, which avoids the need for repeated hospital visits. Given these prescribing patterns, our cost-effectiveness analysis focuses on patients who received NRM/RTV, as they represent the subgroup that could have been treated with either therapy, allowing for a meaningful economic comparison. This approach aligns with real-world clinical decision-making and highlights potential opportunities for cost savings.

An interesting finding from the sensitivity analysis revealed an opposite trend for efficacy, potentially influenced by the different patient selection processes in the two study designs. In RCTs, patient selection is more rigorous, ensuring that the sample meets all eligibility criteria, while real-world studies have less control over patient characteristics, possibly including patients with varying, usually worse, health statuses. For instance, vaccination status could significantly impact efficacy outcomes. In fact, the different results of the sensitivity analysis may be due to the lack of vaccinated patients in the RCTs, which are present in observational studies. The observational study of Ganatra et al. (2023) showed a 60% reduction in relative risk reduction in the hospitalisation for any cause in the NRM/RTV group compared to placebo. Instead, for RDV the data for vaccinated patients are much more limited (Andrews et al., 2024). Due to the potential different efficacy and safety of these drugs among different patients, these two antiviral therapies cannot be prescribed to every patient with a mild-to-moderate form of COVID-19. In particular, NRM/RTV has many drug interactions. Thus, NRM/RTV is not recommended for patients on polytherapy, while only RDV can be administered to patients with chronic kidney disease (Cheng et al., 2022; Gulick et al., 2024). To better apply these findings, sub analyses across different patient populations are recommended, allowing for the selection of the most cost-effective therapy tailored to individual patient characteristics.

Cost analysis indicated that from a healthcare perspective, treating 1,000 patients with NRM/RTV could result in savings of €550,854.46 compared to RDV. Both antiviral therapies demonstrate improved efficacy when administered within 48 h of symptom onset, which could further enhance clinical outcomes and cost-effectiveness by reducing hospitalizations (Andrews et al., 2024). However, the included studies did not provide sufficient data to conduct a sensitivity analysis specifically evaluating this time-dependent effect within our network meta-analysis. Given the significant implications of early treatment initiation, future research should explore the potential benefits of administering antivirals within the first 48 h of diagnosis, assessing both clinical efficacy and economic impact to better inform healthcare decision-making. The limitations of this analysis are the absence of a societal cost perspective, costs to clinics such as scheduling patients on short notice, and opportunity costs, which would likely further favour NRM/RTV due to differences in administration settings. RDV requires a 3-day outpatient treatment, where both the patient and the caregiver would spend at least 1 h per day receiving drug administration, as estimated in the time-flow analysis (Supplementary Table S1). This outcome will be reviewed in the future if oral RDV will be available (McCarthy, 2023).

Overall, the early treatment interventions had a significant positive impact on healthcare resources, as shown by Pierre et al. (2023). However, in the comparison between early treatments for COVID-19 the budget impact analysis supports NRM/RTV, with several limitations preventing a longer-term estimate. One key uncertainty is the upcoming price renegotiationing for both antivirals in 2025, which could significantly alter the cost comparison between these treatments. Additionally, the future number of patients expected to receive these therapies was approximated due to limited data. Specifically, the number of vulnerable patients eligible for treatment in the examined region was unavailable, and historical data from the early period when these therapies were first indicated are skewed by health policies aimed at containing SARS-CoV-2, which are no longer in place. Furthermore, the early COVID-19 treatments prescribed by primary care physicians—a smaller subset compared to those prescribed by specialists—were not accounted for in this analysis. Another limitation is that the cost analysis was performed from an Italian healthcare perspective using Italian salaries and prices. While our budget impact analysis was conducted within the Italian healthcare system, the methodology can be applied to other settings by adjusting cost parameters such as personnel salaries, hospitalization expenses, and drug pricing to reflect local healthcare conditions.

In order to decide which one is better, a network meta-analysis with a larger number of studies should be performed, possibly with a direct comparison of the two drugs. A limitation of our study was that we not consider the indirect costs of treatment, which could have contributed to a better cost analysis. Another factor that could have made the analysis more accurate was to categorise the costs of serious adverse events by type of adverse event. Furthermore, the outcomes of therapies among the different eligible patient targets must be analysed more specifically to implement a more accurate choice in clinical practice and a better evaluation of adverse events costs. In conclusion, to choose which antiviral therapy to administer, physicians should consider the prescribing indications, which limit the use of NRM/RTV more than RDV due to its high rate of interactions with other drugs, and in cases where early therapy can be chosen for COVID-19, NRM/RTV is currently the best choice in economic terms.

## Data Availability

The original contributions presented in the study are included in the article/Supplementary Material, further inquiries can be directed to the corresponding author.
