# Supplementary figures and images for: Efficacy and safety of antiviral treatments for symptomatic COVID-19 outpatients: network meta-analysis and budget impact analysis
Source: Front Pharmacol. 2025 Apr 16;16:1537018. doi: 10.3389/fphar.2025.1537018 (PMC12041651; doi:10.3389/fphar.2025.1537018)

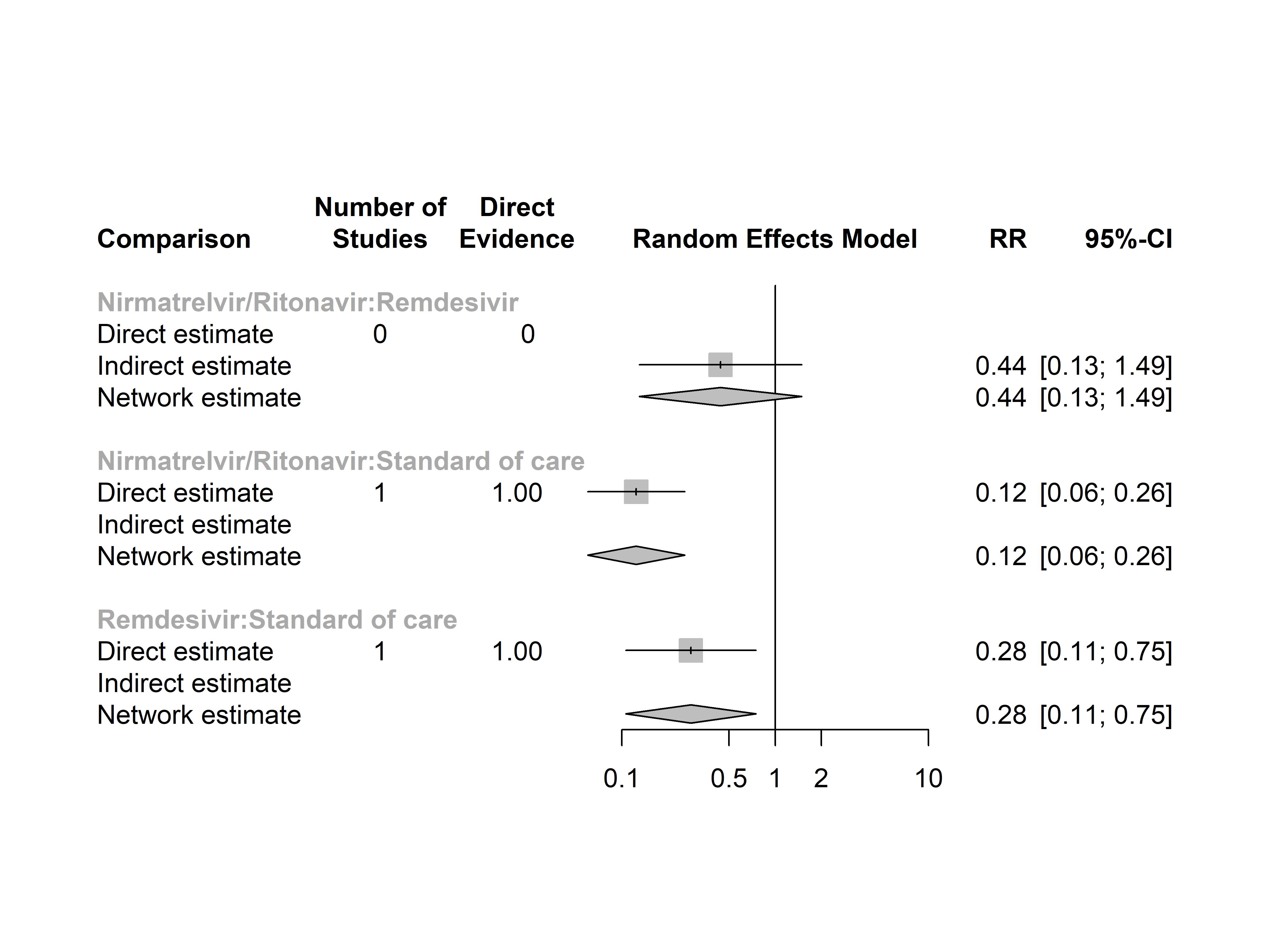

Supplement: Supplementary file 1 [file Image3.jpeg]

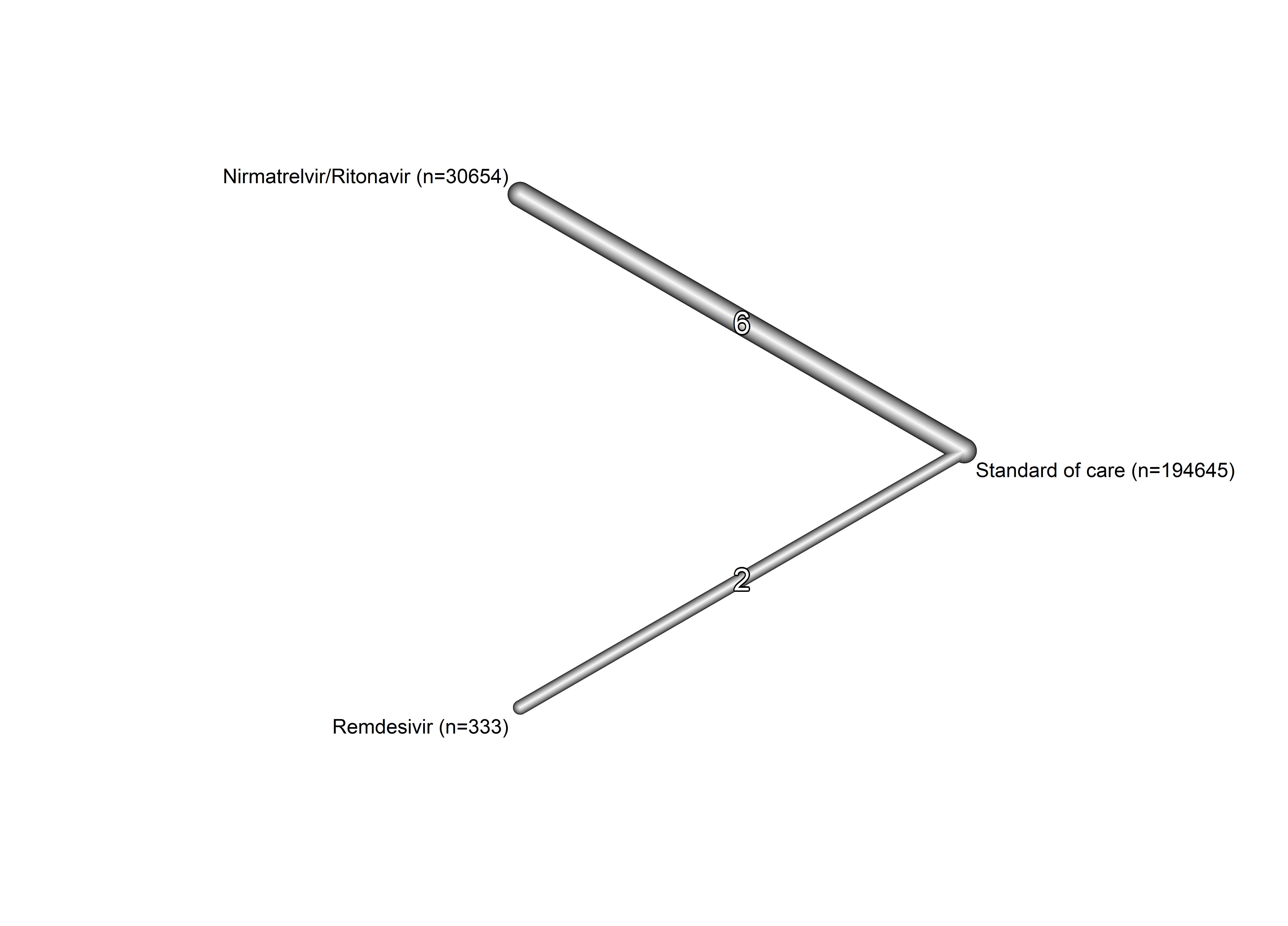

Supplement: Supplementary file 3 [file Image1.jpeg]

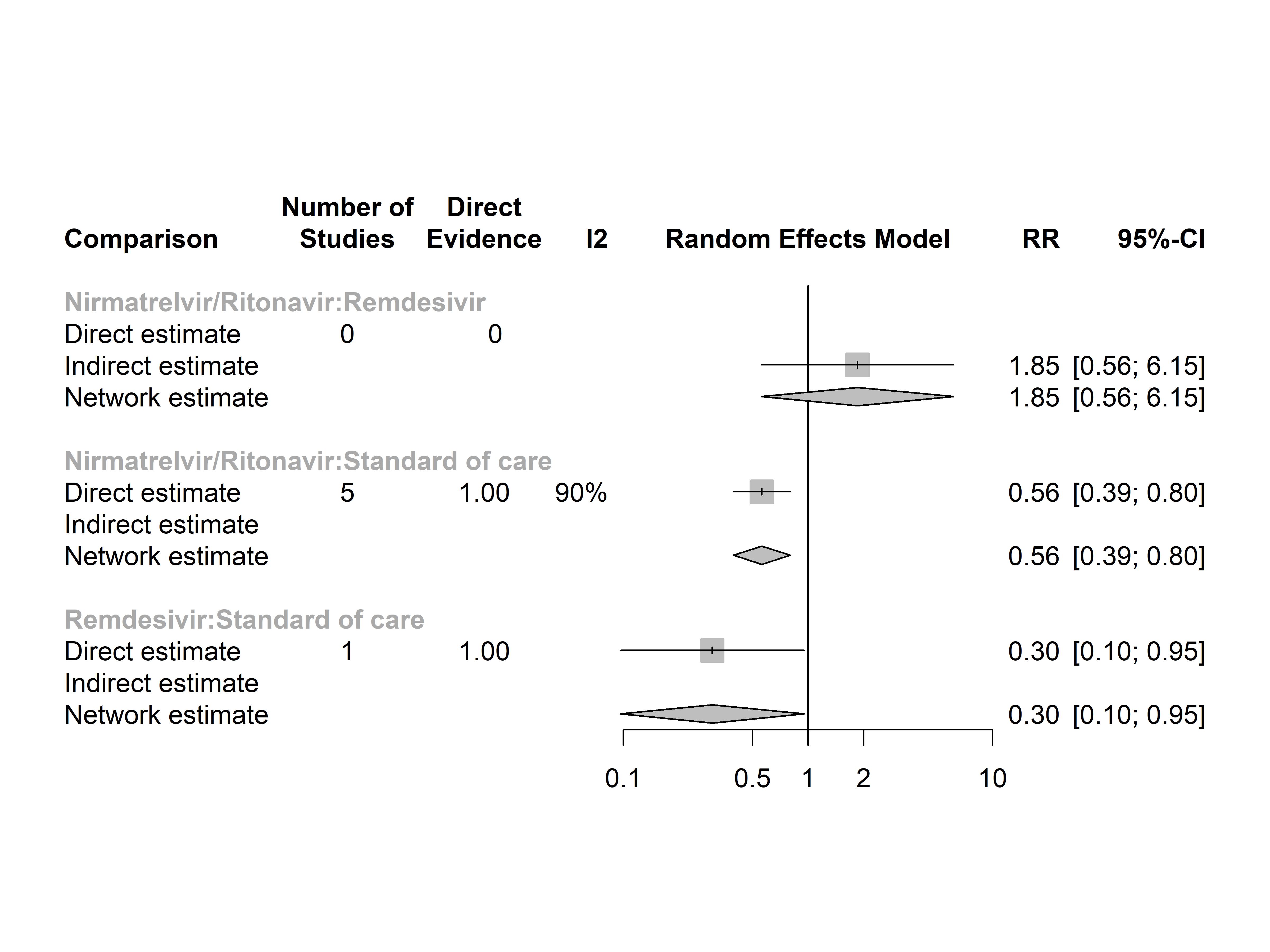

Supplement: Supplementary file 4 [file Image4.jpeg]

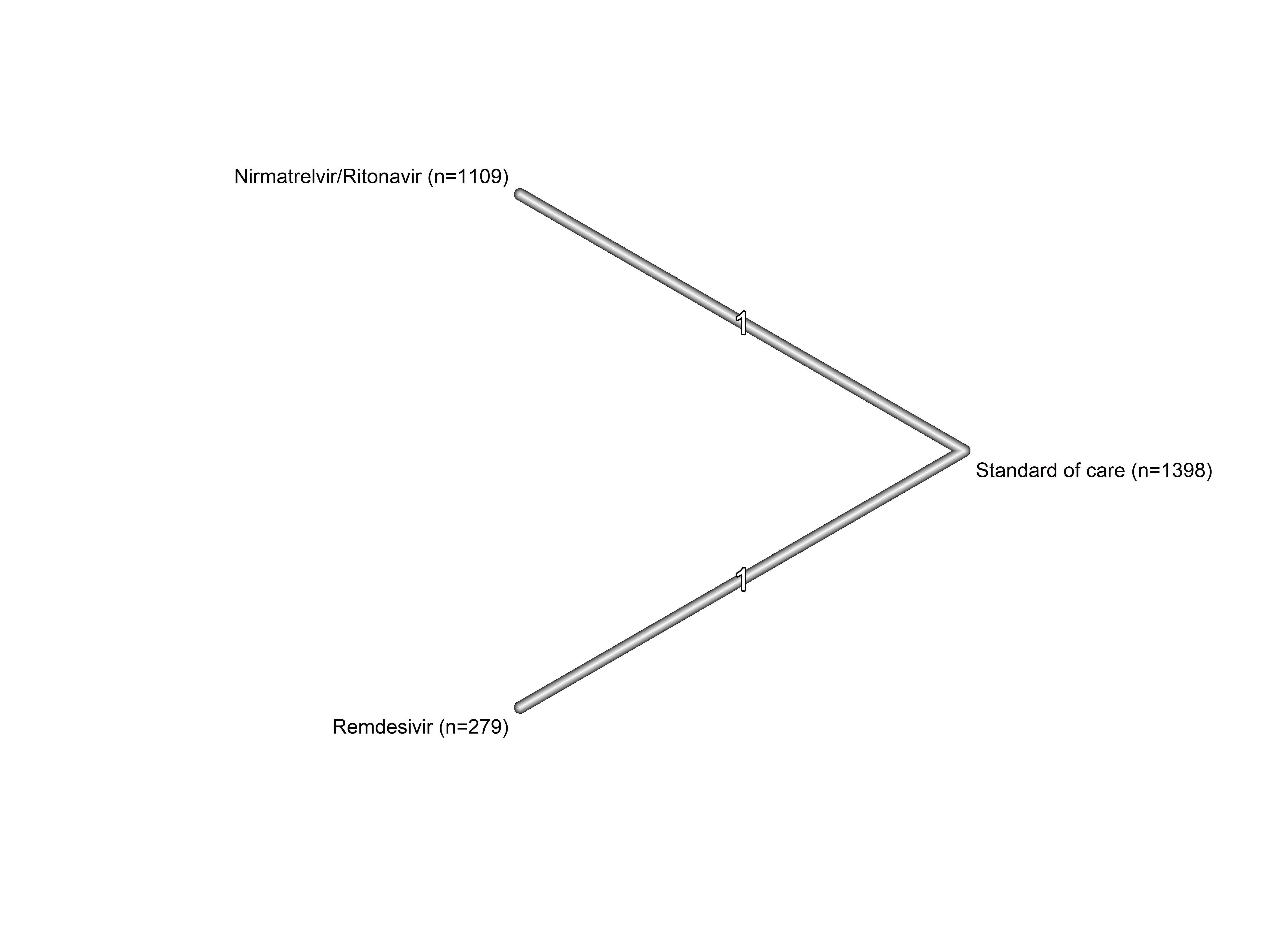

Supplement: Supplementary file 5 [file Image2.jpeg]

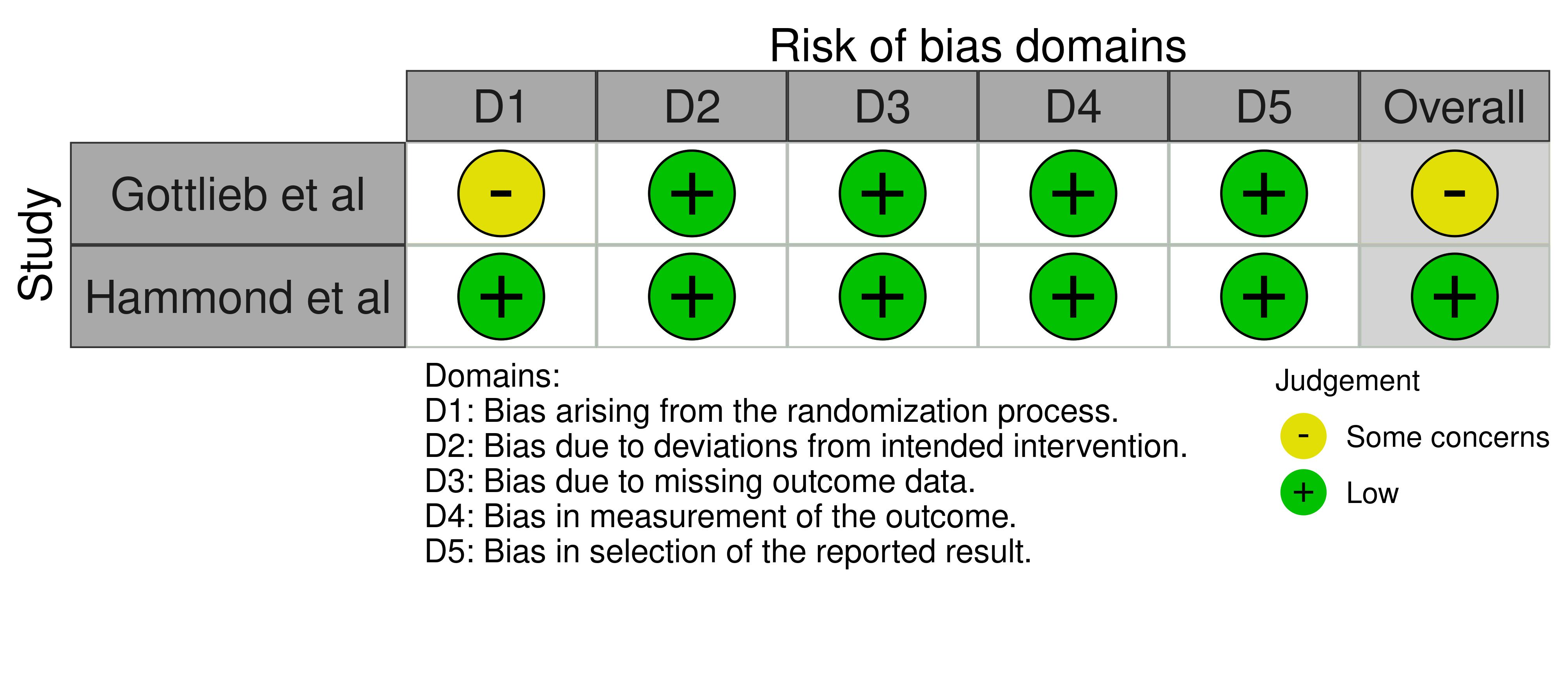

Supplement: Supplementary file 6 [file Image5.jpeg]
