# Supplementary material for: Efficacy and safety of antiviral treatments for symptomatic COVID-19 outpatients: network meta-analysis and budget impact analysis
Source: Front Pharmacol. 2025 Apr 16;16:1537018. doi: 10.3389/fphar.2025.1537018 (PMC12041651; doi:10.3389/fphar.2025.1537018)
Supplement: Supplementary file 2 [file Table1.docx]

Table S1

Table S1 Time flow administration for remdesivir. The duration of each phase was estimated by a panel of pharmacists at University Hospital of Padua based on actual experience following the therapeutic indications.

| **Phase** | **Activity** | **Personnel** | **Time (min)** |
| --- | --- | --- | --- |
| Patient preparation | Reception | Secretary | 5 |
|  | Preparation patient | Nurse | 5 |
|  |  | Physician | 10 |
| Drug preparation | Prescription check | Pharmacist | 2 |
|  | Preparation of parenteral infusion | Nurse | 5 |
|  | Final check of parenteral infusion | Pharmacist | 2 |
| Drug administration | Preparation of materials for parenteral infusion | Nurse | 5 |
|  | Administration | Nurse | 30 |
