# Supplementary material for: Efficacy and safety of antiviral treatments for symptomatic COVID-19 outpatients: network meta-analysis and budget impact analysis
Source: Front Pharmacol. 2025 Apr 16;16:1537018. doi: 10.3389/fphar.2025.1537018 (PMC12041651; doi:10.3389/fphar.2025.1537018)
Supplement: Supplementary file 7 [file Table2.docx]

Table S2

Table S2 Health care professional hourly cost

| **Personnel** | **Hourly cost (€/h)** |
| --- | --- |
| Physician | 45.90 |
| Pharmacist | 39.13 |
| Nurse | 18.06 |
| Secretary | 15.25 |

Data sources were (reference).
