# Supplementary material for: Efficacy and safety of antiviral treatments for symptomatic COVID-19 outpatients: network meta-analysis and budget impact analysis
Source: Front Pharmacol. 2025 Apr 16;16:1537018. doi: 10.3389/fphar.2025.1537018 (PMC12041651; doi:10.3389/fphar.2025.1537018)
Supplement: Supplementary file 8 [file Table3.docx]

Table S6 Risk of bias of observational studies

| **Study** | **Selection** | **Comparability** | **Outcome** | **NOS score** | **AHRQ** |
| --- | --- | --- | --- | --- | --- |
| Arbel et al | 4 | 1 | 3 | 8 | Good quality |
| Ganatra et al | 3 | 2 | 3 | 8 | Good quality |
| Ka Chung Wai et al | 4 | 1 | 3 | 8 | Good quality |
| Lopez et al | 4 | 2 | 3 | 9 | Good quality |
| Wong et al | 4 | 2 | 3 | 9 | Good quality |
| Yip et al | 4 | 1 | 2 | 7 | Good quality |

Score is represented by points in each domain (Selection, Comparability and Outcome). Total score ranges from zero to nine. NOS- Newcastle-Ottawa scale, AHRQ- Agency for Healthcare Research and Quality.
